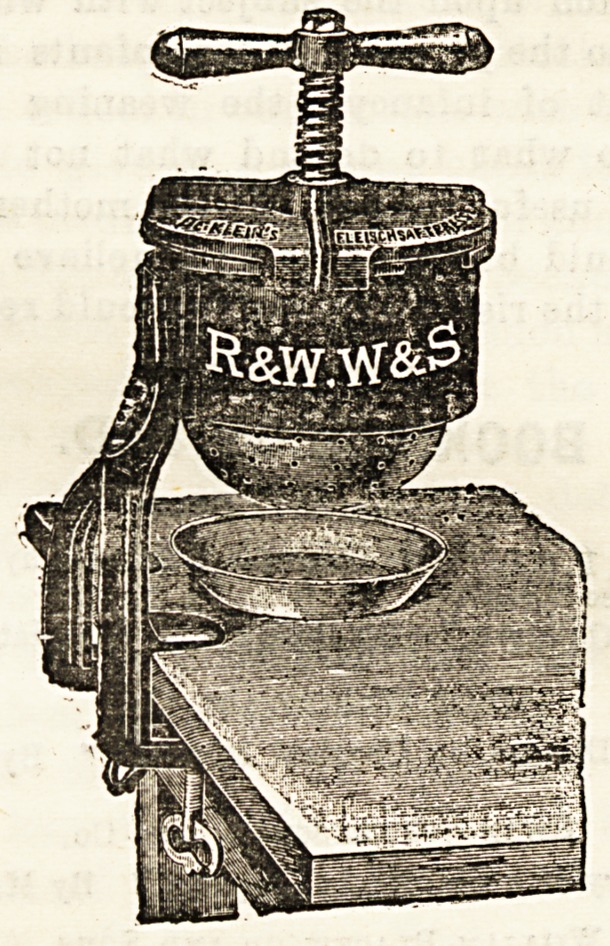# New Appliances and Things Medical

**Published:** 1899-01-14

**Authors:** 


### NEW APPLIANCES AND THINGS MEDICAL.

**[We shall be glad to receive, at our Office, 28 & 29, Southampton Street, Strand, London, W.C., from the manufacturers, specimens of all new preparations and appliances which may be brought out from time to time.]**

### MEAT JUICE EXTRACTOR.

(R. AND W. WILSON AND SONS, 90, WARDOUR STREET, W.)

THERE is a great deal to be said in favour of using freshly-prepared food, especially when it is a matter of giving nourishment of a kind which will not keep. Notwithstanding the immense improvements which late years have seen in the preparation and preservation of meat extracts, juices, jellies, and many other forms of animal food, preparations which from their handiness and from their being constantly ready at any moment are of the greatest service in the sick-room, there undoubtedly are drawbacks in many of them. A something of the nature of which we know but little is apt to be lost in the process of preparation, as an instance of which we may mention the well-known antiscorbutic property of fresh meat, a property which is shared in but to a small degree by many meat preparations in the market. Physicians often order the freshly-prepared juice of meat for their patients, and no doubt would do so much more frequently than they do but for the known difficulties which stand in the way of its preparation. Hence the advantage of such an instrument as that to which we now draw the attention of our readers. The accompanying plate is sufficient to show that the apparatus is a forcible screw-press, by means of which the meat can be so squeezed as to force the juice out of it. The press has a clamp by which it can be firmly fixed to a dresser or a kitchen table, and all the parts which come in contact with the meat can be taken to pieces for cleaning. The apparatus is very strongly made and is thoroughly effective.

### SOLUBLE COCOA.

(ABRAM LYLE AND SONS, LIMITED, 21, MINCING LANE, E.C.)

THIS soluble cocoa appears of excellent quality and exceedingly pleasant flavour. Its high degree of solubility appears to have been obtained without any sacrifice of purity, and we recommend it to the notice of those who can appreciate that which is best in this kind of innocent beverage.

### OLLAPEARL DENTIFRICE.

(OLLAPEARL COMPANY, 371, OXFORD STREET, W.)

A SAMPLE of this new dentifrice has been submitted to us for examination. It has good cleansing properties, is highly fragrant, and pleasant to use, results which are achieved without the use of powerful or injurious chemical ingredients. The box in which the dentifrice is supplied is very ingenious and practical—a small reservoir at one end adapted to the brush head is supplied from a stock compartment with just sufficient powder for each time of using; waste and excess in quantity are thus avoided.

## Figures and Tables

**Figure f1:**